# CYCLANDS: Cycling geo-located accidents, their details and severities

**DOI:** 10.1038/s41597-022-01333-2

**Published:** 2022-05-26

**Authors:** Miguel Costa, Manuel Marques, Carlos Roque, Filipe Moura

**Affiliations:** 1 Civil Engineering Research and Innovation for Sustainability, Instituto Superior Técnico, Universidade de Lisboa, Av. Rovisco Pais, 1, 1049-001 Lisboa, Portugal; 2 Institute for Systems and Robotics, Instituto Superior Técnico, Universidade de Lisboa, Av. Rovisco Pais, 1, 1049-001 Lisboa, Portugal; 3 Laboratório Nacional de Engenharia Civil, Departamento de Transportes, Núcleo de Planeamento, Tráfego e Segurança, Av. do Brasil 101, 1700-066 Lisboa, Portugal

**Keywords:** Civil engineering, Research data

## Abstract

Several cities and national authorities across the globe publish records on road accidents and crashes. This data is vital for road safety analysis, enabling researchers to develop models to understand how different factors impact the frequency and severity of accidents. However, researchers studying cycling safety face additional challenges as datasets containing solely cycling accidents are scarce, may contain errors, among others. Thus, we publish CYCLANDS: CYCling geo-Located AccideNts, their Details and Severities. CYCLANDS is a curated collection of 30 datasets on cycling crashes to lower the barrier in objective cycling research comprising nearly 1.6 M cycling accidents. All observations include the severity and location of the accident. This collection fosters the worldwide study of cycling safety by providing a testbed for researchers to develop tools and models for cycling safety analysis, ultimately improving the safety of those who cycle.

## Abstract (summary)

**Table:** 

Measurement(s)	Cycling Accident
Technology Type(s)	Reports
Sample Characteristic - Location	World

## Background & Summary

Cycling safety research seeks to understand how safe cycling is and what factors related to the individual, the bicycle, and the surrounding environment influence cycling safety. Its research is vital because it envisages a safer environment for everyone to cycle, aiming to decrease the number of accidents and decrease the severity of each accident. More, it is increasingly important to address the cycling safety issue better, potentially through infrastructure design and other interventions, as safety concerns greatly discourage people from cycling^1–3^. Thus, improving the safety of cyclists is a crucial component in any cities’ strategy that seeks to increase cycling numbers^3,4^, while also protecting those who cycle.

Cycling safety is often measured in terms of the number of injuries or fatalities suffered by cyclists. It is often recorded on police reports or hospital admissions. However, most incidents are often not reported (or underreported)^5,6^, resulting in underestimates in gauging how (un)safe cycling is. Nevertheless, accident or crash records are vital because they form the basis for cycling safety research. Researchers use these cycling accidents records for analyzing how factors such as demographic data, built environment, weather, and behavior increase or decrease the risk cyclists face of being involved or injured in an accident^7^. For instance, accident records act as the foundation for accident frequency and severity models where researchers analyze and quantify which factors impact the outcome of accidents and what measures should be taken to protect cyclists.

Fortunately, there is a growing number of cities that are publishing road accident data. However, this data is often published for all transport modes, making it difficult for researchers who only want to focus on vulnerable road users and the particular case of cyclists. Not only this, but cycling safety researchers face many other challenges when working with the provided data. First, accident data is often fragmented into different files, where distinct files contain location information, anonymized personal characteristics, involved vehicle attributes, weather, and road conditions, among others. All different files must be compiled and merged to account for all the accident attributes captured. Second, data specifications differ for each authority publishing the dataset, with even injury severity levels specific to a single country or region. The third and final main challenge is that the published data often contain errors (e.g., accident coordinates wrongly located). Thus, researchers ought to perform a series of validation steps before even being able to study what makes an accident happen. Furthermore, all these challenges also hamper the comparison and transferability of models on cycling safety from one location to another, hindering knowledge transfer from one location to the other.

**Fig. 1:**
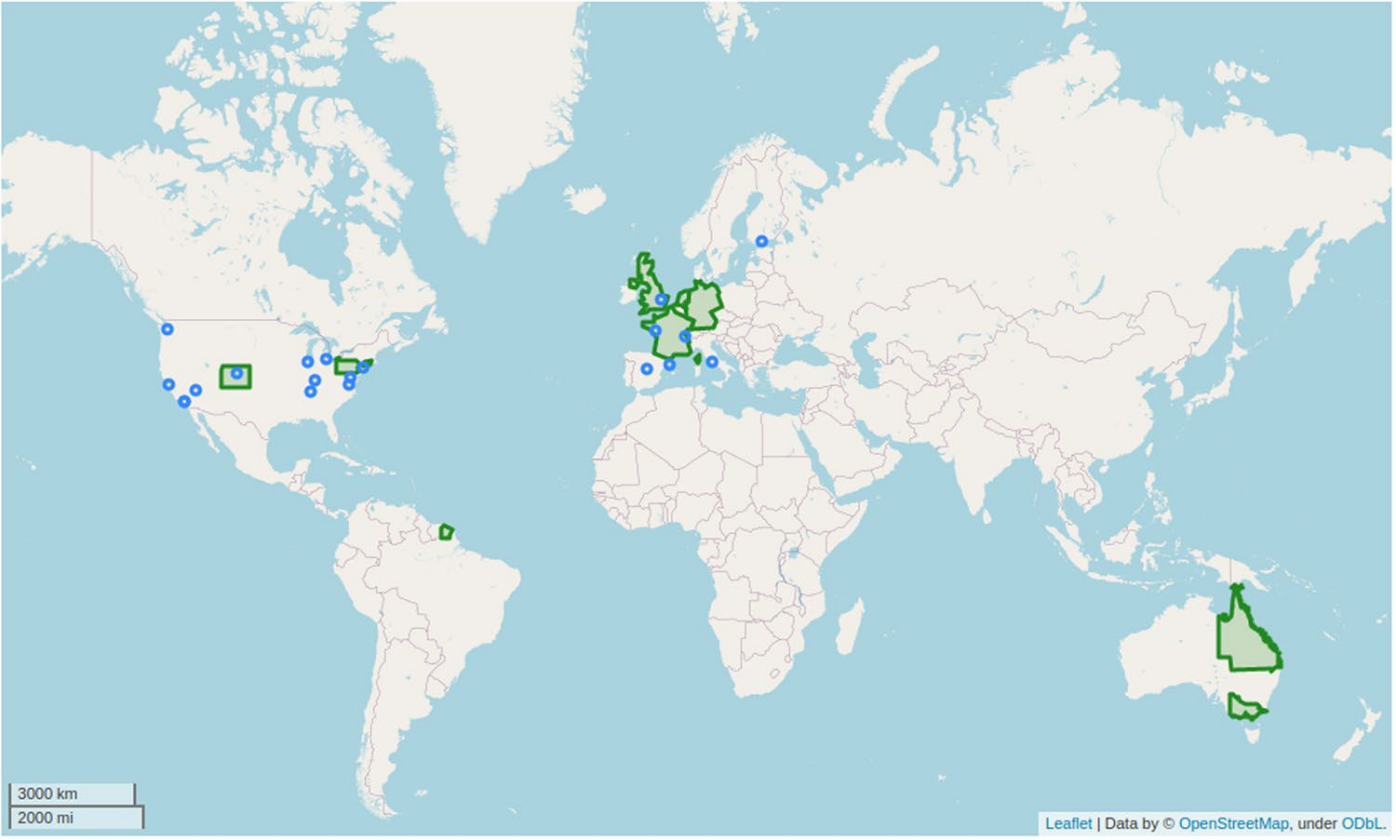
Location of the collected datasets. Datasets range from city level (in blue) to region or country-wide (in green) cycling accident observations.

Hence, validated datasets are required to lower the barrier of cycling safety research. To the best of our knowledge, such an extensive compilation of curated cycling accident records is non-existent today. Thus, we publish CYCLANDS: CYCling geo-Located AccideNts, their Details and Severities. CYCLANDS is a collection of 30 datasets on objective cycling safety (i.e., based on accidents and crashes counts), comprising 1.58 M cycling accident records with geographical scales ranging from country, region, or city-level data. The location of these datasets is laid out in Fig. 1. We present this data in an easy-to-access CSV format, along with the code used for curating and validating each dataset from its source. We expect this data to be particularly beneficial for researchers working with severity models (such as discrete outcome models) or similar. There is a need for a worldwide effort concerning improvement and standardization in crash data collection to monitor the evolution of cycling safety plans and policies and implement the most effective safety countermeasures. Our collection provides a solid contribution to fulfill this need.

This collection of cycling accident records aims to facilitate the comparison of research, tools, and models on cycling safety. Each dataset was individually gathered, curated, and validated to create a testbed for researchers. We aim to promote further the study and understanding of how, what, and why the risk faced by cyclists is still too high. Additionally, we have published this collection on an online site for anyone to visualize and explore the pervasiveness of cycling accidents. Potentially, this site can promote the usage of the data and foster research to protect cyclists further.

## Methods

Cycling accident data form the basis for any research on cycling safety. We begin by searching for publicly available data on road accidents (including accidents involving cars, cyclists, pedestrians, and other road users). After downloading the original data, we filter the collected datasets, resulting in geo-located observations with severity outcomes accidents where any number of cyclists are involved. We further curate and validate the collected data to ensure that the corresponding datasets are easy-to-use and researchers do not need much validation work, essentially lowering the barrier on cycling safety analysis.

### Downloading data

To make a collection of cycling accident data, we began by searching for traffic accident data, as national authorities and cities more commonly publish these. Traffic accidents include observations of accidents involving all road users: cars, public transportation, pedestrians, and cyclists, among others. We focused on looking for data from cities with different sizes, distinct continents, with different cycling modal shares, and with different cycling maturities. However, our final collection was greatly influenced by the data availability, as most cities worldwide do not yet publish any data or statistics on traffic safety. Thus, we included and downloaded data for different geographic scales (ranging from city, region, or country-wide data) and for which their licensing or usage conditions do not hinder its usage for research purposes. Hence, to the best of our knowledge, at this point, all datasets in this collection are available under the public domain, allow for reuse for scientific purposes, or have no licensing terms. Table 1 enumerates all 30 datasets in our collection, alongside some basic information for each, including the number of cycling accidents.

**Table 1:** Datasets in the collection.

Location	Source	Dataset Size	Cycling Sample		Dataset Dates	Geog. Scale	License
Barcelona, Spain	^10^	86701	5144	(5.93%)	2010–2018	City	CC BY 4.0
Cambridgshire, UK	^11^	11954	2647	(22.14%)	2012–2017	Region	Open Gov. Licence (OGL)
Chicago, USA	^12^	384200	5784	(1.51%)	2013–2020	City	Chicago Data Terms of Use
Colorado, USA	^13^	995203	11192	(1.12%)	2004–2018	State	CC BY 3.0
Connecticut, USA	^14^	18169	18169	(100%)	1995–2020	State	Research, informational purposes
Denver, USA	^15^	176958	2298	(1.3%)	2013–2020	City	CC BY 3.0
Detroit, USA	^16^	214469	1477	(0.69%)	2009–2018	City	Unspecified
France	^17^	958471	24813	(2.59%)	2005–2018	Country	Licence Ouverte/Open Licence
Genebra, Switzerland	^18^	25493	1792	(7.03%)	2010–2018	City	SITG Open Data
Germany	^19^	827140	215566	(26.06%)	2016–2019	Country	DL-DE-> BY-2.0
Helsinki, Finland	^20^	48101	3078	(6.4%)	2000–2018	City	CC BY 4.0
Las Vegas, USA	^21^	37086	363	(0.98%)	2015–2017	City	City of Las Vegas Custom License
Los Angeles, USA	^22^	520699	18190	(3.49%)	2010–2020	City	Unspecified
Louisville, USA	^23^	255541	1273	(0.5%)	2010–2017	City	Other (Public Domain)
Madrid, Spain	^24^	304805	6365	(2.09%)	2010–2019	City	Condiciones de Uso
Nantes, France	^25^	7551	1851	(24.51%)	1998–2018	Region	Licence Ouverte v2.0 (Etalab)
Nashville, USA	^26^	296826	773	(0.26%)	2010–2020	City	Public Domain
Netherlands	^27^	1070263	150678	(14.08%)	2003–2018	Country	Unspecified
New York, USA	^28^	1674 490	44384	(2.65%)	2013–2019	City	NYC Open Data Terms of Use
Pasadena, USA	^29^	17027	739	(4.34%)	2008–2017	City	Request permission to use
Pennsylvania, USA	^30^	2596 801	29742	(1.15%)	1999–2018	State	Unspecified
Queensland, Australia	^31^	328247	14747	(4.49%)	2001–2018	State	CC BY 4.0
Richmond, USA	^32^	492	492	(100%)	2009–2015	City	Public Domain
Roma, Italy	^33^	1093040	3933	(0.36%)	2006–2019	City	CC BY 4.0
San Jose, EUA	^34^	584085	17701	(3.03%)	1977–2021	City	Unspecified
Seattle, USA	^35^	201549	5666	(2.81%)	2005–2020	City	PDDL 1.0
UK (Collideoscope)	^36^	100053	100053	(100%)	2013–2020	Country	Unspecified
UK (.gov)	^37^	8394089	892644	(10.63%)	1979–2018	Country	Open Gov. License (OGL)
Victoria, Australia	^38^	1358	51	(3.76%)	2016–2019	State	CC BY 4.0
Washington DC, USA	^39^	222087	3978	(1.79%)	2009–2020	City	CC BY 4.0

Source of the dataset, original dataset size, size of the cycling sample, the dataset date, its geographical coverage and scale, and the license for each dataset are reported.

We searched traffic accident data in different platforms, such as cities’ open data portals and maps, national statistics bureaus, and national open data platforms. Typically these platforms aggregate the data collected by police authorities and hospitals about traffic accidents, their locations, the severity outcomes, and the conditions in which the accident happened. After reviewing the data to ensure that it could be reused for research purposes, it was downloaded in Excel, CSV, GeoJSON, or any other format.

### Cycling data filtering

Next, after downloading the datasets, we filter the data such that in the end, we have accidents where cyclists were involved. This step is needed because cities’ accident and collision data include accidents for all transport modes (motorized vehicles, bicycles, and pedestrians). Since most cycling safety research rely solely on cycling observations or accidents where cyclists were involved, we filter the original dataset only to have observations concerning bicycles.

For that, we scrutinize each observation to identify whether a bicycle or cyclist was involved in the accident. We scout such information from the user or vehicles involved, matching cases for which information is scattered across several sources. If a positive match is found, the observation is filtered and added to the final cycling dataset.

### Data curation

The third and final step in our process consists of curating the cycling accident data. Once we filter the cycling data, we curate the data, ensuring that the data can be easily handled by any researcher and consistent across the collection. Table 2 shows some information about the contents of each dataset.

**Table 2:** Summary of information available in each dataset of this collection.

Dataset Location	# of Outcome Classes^1^	Light Conditions	Road Conditions	Weather Conditions	Personal Features	Vehicle Features	Latidude/Longitude
Barcelona, Spain	4				✓	✓	✓
Cambridgshire, UK	4	✓	✓	✓	✓	✓	✓
Chicago, USA	3	✓	✓		✓	✓	✓
Colorado, USA	5	✓	✓	✓	✓	✓	✓
Connecticut, USA	5	✓	✓	✓		✓	✓
Denver, USA	3	✓	✓		✓	✓	✓
Detroit, USA	5	✓	✓	✓		✓	
France	4	✓	✓	✓	✓	✓	✓
Genebra, Switzerland	4	✓	✓	✓		✓	✓
Germany	4	✓	✓			✓	✓
Helsinki, Finland	3						✓
Las Vegas, USA	6	✓	✓	✓	✓	✓	
Los Angeles, USA	3				✓	✓	✓
Louisville, USA	3	✓	✓	✓		✓	✓
Madrid, Spain	3		✓	✓	✓		
Nantes, France	4						✓
Nashville, USA	3	✓		✓		✓	✓
Netherlands	3			✓	✓	✓	✓
New York, USA	3					✓	✓
Pasadena, USA	3	✓	✓	✓	✓	✓	✓
Pennsylvania, USA	5	✓	✓	✓	✓	✓	✓
Queensland, Australia	3	✓	✓	✓		✓	✓
Richmond, USA	1						✓
Roma, Italy	3	✓	✓		✓	✓	✓
San Jose, EUA	5	✓	✓	✓	✓	✓	✓
Seattle, USA	4	✓	✓	✓		✓	✓
UK (Collideoscope)	4						✓
UK (.gov)	4	✓	✓	✓			✓
Victoria, Australia	1				✓		✓
Washington DC, USA	5						✓

The number of outcome (severity) classes of the accident in each dataset is reported, along whether other types of information are also disclosed.

^1^Outcomes vary per datasets and include some of the following classes: Property Damage Only, Injury, Serious Injury, Fatality, and others.

We begin by curating the datasets based on the geographical location of accidents. Knowing the location of accidents is vital in safety research. Many use this information as the foundation to analyze the built environment in such locations and how it impacts accidents. Although all added datasets include the location to some degree, the description of accident locations varies greatly across datasets. Accident locations on each dataset can be detailed in one of four formats. Some describe locations based on geographic coordinates under the WSG 84 coordinate system, while others use specific coordinate systems or projections for the specific referenced region. Other datasets’ accident locations are not as simple or accurate, and crashes are instead located with an address, the closest intersection to the location, or a description of the location (i.e., 200 meters west of the intersection of streets A and B). Given the importance of crash locations, we standardize when feasible crash locations to a single format, which we chose to be the WSG 84 coordinate system given its wide use across many applications worldwide. Thus, for datasets whose crash locations are in a projection system different from the WSG 84, we automatically project all accident locations to the WSG 84 coordinate system. This projection was applied to the following datasets, where the original coordinate system is detailed in parentheses: Barcelona (UTM), Helsinki (EPSG:3879), Netherlands (EPSG:28992), Queensland (GDA94), and UK (.gov) (OSGR) datasets. For the datasets which do not contain any geographic coordinates and are instead located using an address or description, no projection is applied (Detroit, Las Vegas, and Madrid datasets). Ultimately, this allows for locations to be more easily discoverable and circumvents researchers’ needs to identify and project the coordinate system of the original dataset for those datasets which use a specific coordinate system to the related area.

Next, we standardize the date and time details of each observation. Knowing when an accident has happened allows researchers and urban authorities to identify the frequency of accidents and any related trends. Consequently, we standardize the format of how accident dates are enumerated across datasets, easing the analysis of accidents over time.

Finally, we filter accident observations to ensure that key fields (accident severity, location, and date) do not contain any erroneous or missing elements. Localized accident severity models are vastly reliant on knowing both the location of accidents and their outcome. With this in mind, we iterate over all observations and filter entries where these variables are either 0, NaN, None, empty, or correspond to an Unknown value. Equally important, observations with incorrect or invalid data in these variables are also filtered. This means that observations with unfeasible locations or annotated outside their respective geographic boundaries are removed (e.g., accidents outside the state of Colorado are removed for the Colorado dataset, or accidents with location [0.0, 0.0] for the Barcelona dataset are also excluded). This step enables the inclusion of only observations which contain critical information for cycling safety research. In the end, we present our collection of cycling accident datasets as presented in the Data Records section.

With this procedure we ensure that all final records have detailed and accurate information about both accidents’ outcomes and where these have happened, along with other accident information. Following a growing need for improving and standardizing crash data collection, this procedure ensures a solid step for monitoring cycling safety data. This, in turn, can help research to design plans and policies and implement the most effective safety countermeasures to help protect cyclists.

## Data Records

For each dataset, we provide the curated data on cycling accidents enumerated in Table 1. We provide a copy of all data at Zenodo^8^, while also making data available in our repository^9^. Additionally, we published an online site (https://ushift.tecnico.ulisboa.pt/cyclands) for easy exploration and interactive visualization of the datasets.

Tables 3 and 4 expands Table 2 by providing more detailed information about the data contained in each dataset. For each dataset, we provide the following files, where {*name*} corresponds to a given dataset:

- cycling_safety_{name}.csv: The data for each dataset is available as a comma separated value (CSV) file to allow easy use and exploration, regardless of the software used. This file contains all variables available for the data, including the accident severity, location, date, among other variables.
- cycling_safety_{name}.geojson: We also provide the data as a GeoJSON file to facilitate the usage of this data in GIS-based software and other mapping tools, by reducing the need for researchers to transform the data into readable formats for such tools.
- cycling_safety_{name}_summary.txt: Contains a summary of the available variables for the dataset, together with some descriptive statistics of each variable.
- cycling_safety_{name}_license.txt: The license or terms of use for each dataset. The details for the license are provided in cycling_safety_{name}_legalcode.txt.
- cycling_safety_{name}_legalcode.txt: The legal code for the license under which the data is provided.

**Table 3:** Information contained in each dataset. Details of accident locations, personal and vehicle characteristics involved, causes of the accident and data and time.

Dataset Location	Location Details							Personal Characteristics			Vehicle Characteristics					Accident Cause			Date & Time Details	
	Lat & Lon	Address	Traffic Control	Speed Limit	Road Type	Intersection Type	Other^1^	Gender	Age	Other^2^	Type	Maneuvers	Collision Point	Direction	Other^3^	Contributing Factors	Human Factors	Vehicle Factors	Date	Time
Barcelona, Spain	✓	✓						✓	✓	✓	✓								✓	✓
Cambridgshire, UK	✓	✓		✓	✓	✓		✓	✓	✓	✓	✓		✓	✓				✓	
Chicago, USA	✓	✓	✓	✓	✓			✓	✓	✓	✓	✓	✓	✓					✓	✓
Colorado, USA	✓	✓			✓	✓				✓	✓	✓	✓		✓		✓		✓	
Connecticut, USA	✓	✓			✓	✓	✓						✓			✓			✓	✓
Denver, USA	✓				✓	✓				✓	✓	✓	✓	✓			✓		✓	✓
Detroit, USA		✓				✓					✓								✓	✓
France	✓				✓	✓	✓	✓	✓	✓	✓	✓	✓						✓	✓
Genebra, Switzerland	✓				✓	✓					✓					✓			✓	
Germany	✓										✓		✓						✓	✓
Helsinki, Finland	✓																		✓	
Las Vegas, USA		✓								✓	✓		✓	✓		✓			✓	
Los Angeles, USA	✓	✓						✓	✓		✓								✓	
Louisville, USA	✓	✓				✓							✓						✓	✓
Madrid, Spain		✓						✓	✓										✓	✓
Nantes, France	✓																		✓	✓
Nashville, USA	✓	✓									✓								✓	✓
Netherlands	✓	✓		✓	✓			✓	✓		✓	✓	✓		✓				✓	✓
New York, USA	✓	✓									✓							✓	✓	✓
Pasadena, USA	✓	✓		✓				✓	✓		✓	✓	✓	✓		✓			✓	✓
Pennsylvania, USA	✓				✓	✓		✓	✓	✓					✓				✓	✓
Queensland, Australia	✓	✓	✓	✓		✓						✓				✓			✓^4^	
Richmond, USA	✓	✓																	✓	✓
Rome, Italy	✓	✓	✓		✓		✓	✓							✓				✓	✓
San Jose, EUA	✓	✓	✓			✓			✓						✓		✓	✓	✓	✓
Seattle, USA	✓	✓				✓									✓				✓	✓
UK (Collideoscope)	✓																		✓	✓
UK (.gov)	✓		✓		✓	✓													✓	✓
Victoria, Australia	✓				✓	✓		✓	✓										✓	
Washington DC, USA	✓	✓																	✓	✓

^1^Other information include information on Annual Daily Traffic, Nearby a School, Traffic Levels.

^2^Other information include Type of Occupant, School Trip, Trip Purpose, Human/Driver Action, or Human Vision.

^3^Other information include Overtaking, Defects, Action, Accident Type, Lights, or Speeding.

^4^Year only.

**Table 4:** Information contained in each dataset. Details light conditions, road conditions and weather conditions available in each dataset.

Dataset Location	Light Condition Details						Road condition Details										Weather Condition Details						
	Daylight	Dawn	Dusk	Dark	Dark Lighted	Dark Unlighted	Surface Conditions								Road Alignment^2^	Surface Type^3^	Clear	Cloudy	Rain	Wind	Fog	Snow	Other^4^
							Dry	Wet	Frost/Ice	Snow	Mud	Slush	Water	Other^1^									
Barcelona, Spain							✓	✓	✓	✓													
Cambridgshire, UK	✓			✓			✓	✓	✓						✓		✓		✓	✓	✓		
Chicago, USA	✓	✓	✓	✓			✓	✓	✓	✓	✓				✓	✓	✓	✓	✓		✓	✓	✓
Colorado, USA	✓	✓	✓		✓	✓	✓	✓	✓	✓	✓						✓		✓	✓	✓	✓	✓
Connecticut, USA	✓	✓	✓		✓	✓	✓	✓	✓	✓		✓			✓		✓	✓	✓	✓	✓		
Denver, USA	✓	✓	✓		✓	✓	✓	✓		✓	✓	✓		✓									
Detroit, USA	✓	✓	✓	✓			✓	✓		✓			✓				✓	✓	✓	✓		✓	
France	✓	✓	✓		✓	✓	✓	✓	✓				✓				✓	✓	✓	✓		✓	
Genebra, Switzerland	✓	✓	✓	✓			✓	✓									✓	✓	✓	✓		✓	
Germany	✓	✓	✓	✓																			
Helsinki, Finland							✓	✓															
Las Vegas, USA	✓	✓	✓		✓	✓											✓	✓	✓	✓			
Los Angeles, USA							✓	✓	✓	✓			✓		✓								
Louisville, USA	✓	✓	✓		✓	✓	✓	✓		✓				✓			✓	✓	✓		✓		
Madrid, Spain																	✓		✓		✓	✓	
Nantes, France																							
Nashville, USA	✓	✓	✓		✓	✓											✓		✓	✓		✓	
Netherlands																	✓		✓	✓	✓	✓	
New York, USA							✓	✓															
Pasadena, USA	✓	✓	✓		✓	✓	✓	✓	✓	✓	✓			✓	✓	✓	✓	✓	✓				
Pennsylvania, USA	✓	✓	✓		✓	✓	✓	✓							✓		✓		✓		✓	✓	✓
Queensland, Australia	✓	✓	✓		✓	✓											✓		✓		✓		✓
Richmond, USA							✓	✓							✓	✓							
Rome, Italy	✓			✓			✓	✓	✓	✓				✓									
San Jose, EUA	✓	✓	✓		✓	✓	✓	✓	✓	✓				✓			✓	✓	✓	✓		✓	
Seattle, USA	✓	✓	✓		✓	✓	✓	✓	✓	✓	✓			✓			✓	✓	✓		✓	✓	
UK (Collideoscope)																							
UK (.gov)	✓				✓	✓														✓	✓		
Victoria, Australia																							
Washington DC, USA																							

^1^Other information include Debris, Gravel, Slippery, Sand, or Oil.

^2^Road alignment refers to the road being Straight, Curve, or On a Grade.

^3^Surface Types may be Concrete, Asphalt, Dirt, Paved, or others.

^4^Other information include Hail, or Smoke.

## Technical Validation

Substantial efforts were undertaken to verify and validate the quality of the collection of datasets presented here. Individual datasets were found, selected, and obtained from reliable sources linked with cities, municipalities, statistical bureaus, or police records. Acquiring data from this set of sources is routinely employed by cycling safety researchers and form the current research literature.

Then, from the original data, a series of automated data validation steps were undertaken to further validate the correctness of each individual dataset. Datasets were individually checked for key missing features (e.g., crash severity outcome or location) and invalid observations were removed from the final curated data. Additionally, accident locations were compared to their expected locations, i.e., accidents from the Barcelona dataset were compared to Barcelona’s geographical borders. Observations with geographical coordinates which lay far from the feasible dataset boundaries (inaccurate or faulty locations) were also removed from the final collection. Finally, we perform a visual sanity check of the data for each dataset. We visualize accident locations using mapping tools and assess whether accident locations seem feasible. The code for all data curation processes is publicly available, enabling further verification by other researchers. In effect, this ensures that researchers can readily use the data available under this collection with the hope of expediting the barrier of cycling safety research.

## Usage Notes

The collection of cycling accident datasets can be analyzed using different tools or software. We provide each dataset in a single CSV file to facilitate data import into different tools or pieces of software, such as Python, R, NLogit, or others, which are typically used in cycling safety research when using discrete outcome modes, ordered probability models or any machine learning approach. Key accident data for each dataset has been normalized so that information like date (Date) and location (Latitude and Longitude) can be easily found and used.

## Data Availability

Together with the collection of cycling safety datasets, we share the code used for curating the datasets. All code has been written for Python3. We present the code under Jupyter notebooks, which provide step-by-step instructions on how each dataset was curated. The code is available under the MIT license (https://opensource.org/licenses/MIT) and is available at https://github.com/U-Shift/cyclands.
